# Enhancement of Immune Functions by *Limosilactobacillus reuteri* KBL346: In Vitro and In Vivo Studies

**DOI:** 10.3390/ijms25010141

**Published:** 2023-12-21

**Authors:** Chanseop Park, Seon Yeong Ji, Hyun Hwangbo, Seung-yeon Shin, Min Yeong Kim, Kiuk Lee, Da Hye Kim, Bo-Ram Cho, Hyesook Lee, Yung Hyun Choi, Hyun Ju You

**Affiliations:** 1 KoBioLabs Inc., Seoul 08826, Republic of Korealeku@snu.ac.kr (K.L.); boramcho@kobiolabs.com (B.-R.C.); 2 Anti-Aging Research Center, Dong-eui University, Busan 47227, Republic of Koreabelieve0402@naver.com (D.H.K.); 3 Department of Biochemistry, Dong-eui University College of Korean Medicine, Busan 47227, Republic of Korea; 4 Department of Convergence Medicine, Pusan National University School of Medicine, Yangsan 50612, Republic of Korea; lhyes0219@pusan.ac.kr; 5 Department of Food and Nutrition, Research Institute of Human Ecology, Seoul National University, Seoul 08826, Republic of Korea

**Keywords:** *Limosilactobacillus reuteri*, probiotics, macrophage, cyclophosphamide, immunity, safety

## Abstract

Lactobacilli have been widely used as probiotics because of their benefits for intestinal health and physiological functions. Among a variety of *Lactobacillus* genera, *Limosilactobacillus reuteri* has been studied for its ability to exert anti-inflammatory functions and its role in controlling metabolic disorders, as well as the production of the antimicrobial compound reuterin. However, the effects and mechanisms of *L. reuteri* on enhancing immune responses in the immunosuppressed states have been relatively understudied. In this study, we isolated an immunomodulatory strain, namely, *L. reuteri* KBL346 (KBL346), from a fecal sample of a 3-month-old infant in Korea. We evaluated the immunostimulatory activity and hematopoietic function of KBL346 in macrophages and cyclophosphamide (CPA)-induced immunosuppressed mice. KBL346 increased the phagocytic activity against *Candida albicans* MYA-4788 in macrophages, and as biomarkers for this, increased secretions of nitric oxide (NO) and prostaglandin E_2_ (PGE_2_) were confirmed. Also, the secretions of innate cytokines (TNF-α, IL-1β, and IL-6) were increased. In CPA-induced immunosuppressed mice, KBL346 at a dosage of 10^10^ CFU/kg protected against spleen injury and suppressed levels of immune-associated parameters, including NK cell activity, T and B lymphocyte proliferation, CD4^+^ and CD8^+^ T cell abundance, cytokines, and immunoglobulins in vivo. The effects were comparable or superior to those in the Korean red ginseng positive control group. Furthermore, the safety assessment of KBL346 as a probiotic was conducted by evaluating its antibiotic resistance, hemolytic activity, cytotoxicity, and metabolic characteristics. This study demonstrated the efficacy and safety of KBL346, which could potentially be used as a supplement to enhance the immune system.

## 1. Introduction

The immune system plays an important role in maintaining body homeostasis by defending against infections and diseases. The body uses innate and adaptive immune systems to detect and eliminate pathogenic invaders that enter through physical barriers. The innate immune response is an immediate and non-specific defense mechanism that involves the activation of macrophages, neutrophils, and natural killer (NK) cells, which further leads to cytokines production, including tumor necrosis factor alpha (TNF-α), interleukin (IL)-1β, and IL-6. The adaptive immune response provides highly specific protection against pathogens. It is characterized by its ability to recognize and remember specific antigens on the surface of pathogens or foreign substances. Gut-associated lymphoid tissue (GALT) is a significant component of the immune system that is located in the gastrointestinal tract and plays a central role in monitoring and responding to substances that enter the digestive system. The research in recent years has highlighted the significant impact of the gut microbiota in maintaining systemic immunity by modulating GALT and other immune organs [1].

As probiotic gut microbiota, certain strains of lactobacilli isolated from human feces are known to modulate the host immune system [2,3,4,5]. *Lactobacillus* has anti-inflammatory functions, reduces chronic inflammation, maintains immune tolerance and balance, and therefore, controls various types of metabolic and autoimmune diseases [6,7,8]. However, studies on the immunomodulatory function of *Lactobacillus* have been rarely reported in the context of improving immune suppression. Some strains of *L. casei* [9], *L. plantarum* [10,11,12], and *L. acidophilus* [13] isolated from various food sources were reported to enhance the depressed immunity by cyclophosphamide in in vivo models. However, the effects were highly strain specific and most of the strains originated from fermented foods.

*Lactobacillus reuteri* (*Limosilactobacillus reuteri*) reside in various human body sites, primarily including the gastrointestinal tract, urinary tract, skin, and breast milk [14]. *L. reuteri* has several beneficial effects on health by preventing and alleviating symptoms of metabolic and inflammatory diseases [15]. It can also withstand various pH environments and employs multiple mechanisms that allow it to inhibit pathogenic microorganisms including the production of the highly potent anti-microbial metabolite reuterin [16,17]. In addition, *L. reuteri* has stimulatory effects on the immune system by increasing T cell development and function [15]. Upon supplementation with *L. reuteri* ATCC 55730, B lymphocyte numbers were increased and promoted increased numbers of CD4-positive T-lymphocytes in the ileal epithelium [18]. There is growing interest in the immune-boosting potential of *L. reuteri*; however, its efficacy is limited to certain strains.

Here, we aimed to evaluate the immune-stimulating ability and safety profile of *L. reuteri* KBL346 isolated from the feces of a 3-month-old infant. In our study, we used murine macrophage cells as an in vitro model to evaluate various factors, such as phagocytic activity, secretions of nitric oxide (NO) and prostaglandin E_2_ (PGE_2_), immunomodulatory cytokines (TNF-α, IL-1β, IL-6), and the phosphorylation and expression of mitogen-activated protein kinase (MAPK) or nuclear factor-κB (NF-κB) protein. Furthermore, we employed a cyclophosphamide (CPA)-induced immunosuppressed mice model as an in vivo model to examine the histological changes in the spleen, the immune-related blood cell counts, the proliferation of lymphocytes, the natural killer cell activity, the CD4- and CD8-positive T cell counts, the cytokines, and the measurement of immunoglobulins in splenocytes and blood serum obtained from the mice.

## 2. Results

### 2.1. KBL346 Increased the Phagocytic Activity of Macrophages

Phagocytosis occurs when phagocytes consume pathogens or foreign substances after physical/chemical barriers are broken. We confirmed the phagocytic activity of RAW264.7 cells with exposure to KBL346.

**Figure 1:**
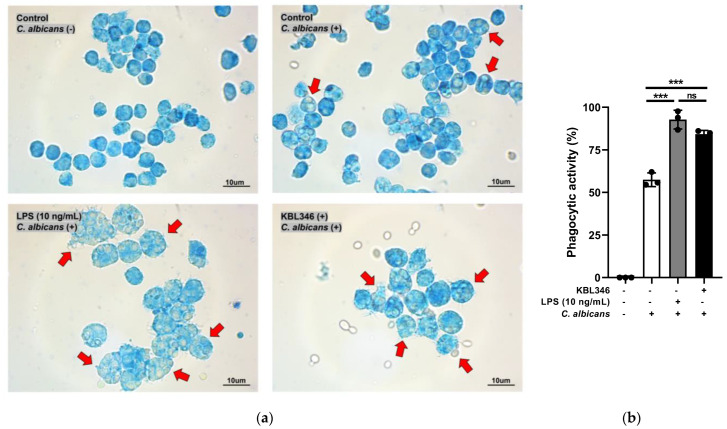
Effects of KBL346 on phagocytic activity in macrophage. After coculture of KBL346 and RAW264.7 cells (ratio, 50:1), *Candida albicans* MYA-4788 was added to RAW264.7 cells at an MOI of 10. (**a**) Light micrograph showing RAW264.7 cells (magnification, ×1000) stained with 0.5% methylene blue reagent. Red arrows indicate phagocytic activities of RAW264.7 cells. (**b**) Phagocytic activity was calculated as the ratio of phagocytic cells to total macrophages in the micrograph. ns, not significant difference compared with the marked sample (*p* > 0.05); * significant difference compared with the marked samples (*** *p* < 0.001).

In the experiment, RAW264.7 cells were cocultured with KBL346, and then *Candida albicans* MYA-4788 was added. The percentage of phagocytic cells among all cells was calculated to measure the phagocytic activity. As depicted in Figure 1, the group treated with KBL346 showed a significant increase in phagocytic activity (up to 84.2%) compared with the group treated with *C. albicans* alone (57.4%). Moreover, the phagocytic activity of the KBL346-treated group was found to be similar to that of the group treated with lipopolysaccharide (LPS), which is a representative immune-stimulating agent (92.8%).

### 2.2. KBL346 Increased Secretion of Nitric Oxide and Prostaglandin Secretion by Macrophages

When a foreign substance enters a phagocyte, it undergoes degradation through various enzymes, reactive oxygen/nitrogen species, and antibacterial peptides within phagolysosomes, which are formed by the combination of phagosomes with lysosomes. Macrophages and neutrophils, which are types of phagocytes, have receptors for C5a and fMLF (fMet-Leu-Phe). When these receptors are activated, the production of reactive oxygen/nitrogen species increases to promote the intracellular destruction of microorganisms within phagolysosomes. In addition, certain types of PRRs initiate inflammasome activation, and activated inflammasomes play a vital role in the induction of inflammatory cascades and coordination of host defenses. Inflammasome activation leads to the release of PGE_2_, which is known to be involved in gut wound repair, although this remains controversial.

**Figure 2:**
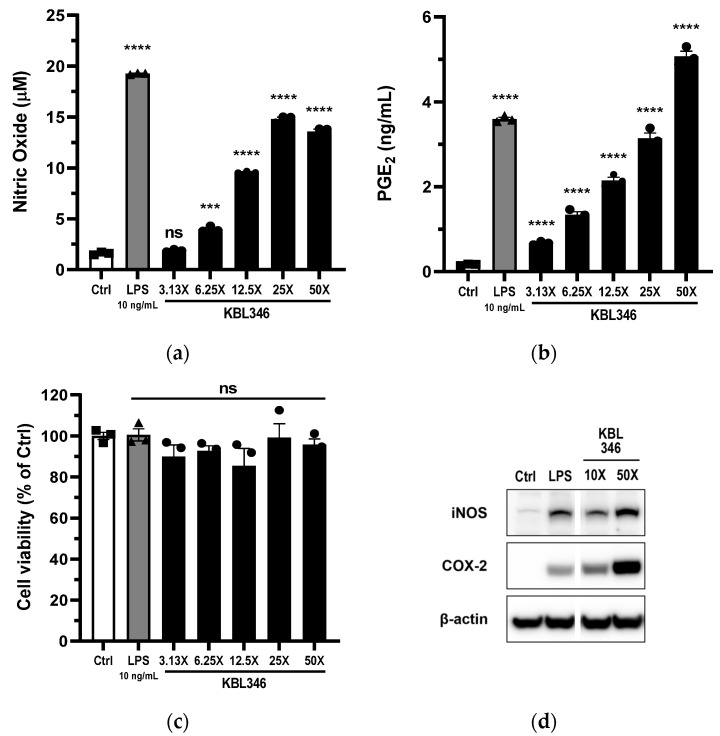
Effects of KBL346 on the production of nitric oxide (NO) and prostaglandin E_2_ (PGE_2_) in macrophages. KBL346 and RAW264.7 cells were cocultured at ratios from 3.13:1 to 100:1. As a positive control group, 10 ng/mL of lipopolysaccharide (LPS) was treated, and the negative control group (ctrl) was not treated with either LPS or bacteria. (**a**,**b**) The levels of immune mediators (NO, PGE_2_) in the culture supernatant of RAW264.7 cells were measured. (**c**) The viability of RAW264.7 cells was assessed using MTT assay. The viability was calculated as a percentage of that in the control group based on the control group using an MTT assay kit. (**d**) After coculturing KBL346 with RAW264.7 cells at ratios of 10:1 and 100:1, the expressions of inducible nitric oxide synthase (iNOS) and cyclooxygenase-2 (COX-2) were analyzed using Western blotting. As a positive control group, treatment with 10 ng/mL of LPS was used. Data are expressed as the mean ± SD (*n* = 3). ns, not significant compared with the control group (*p* > 0.05); * significant difference compared with the control group (*** *p* < 0.001, **** *p* < 0.0001).

We used ELISA to measure the levels of NO and P PGE_2_ in culture supernatants, and Western blotting to determine the expression of iNOS and COX-2, which are responsible for the production of NO and PGE_2_. When we added KBL346 to RAW264.7 cells, we observed an increase in the secretion of NO and PGE_2_ (as shown in Figure 2a,b). There was no reduction in cell viability at any of the concentrations tested, as seen in Figure 2c. Treatment of cells with KBL346 (ratio, 50:1) resulted in a significant increase of up to 9.25-fold (14.8 μM) in the secretion of NO compared with the control group (1.6 μM).

The amount of PGE_2_ secretion was significantly higher in the group treated with KBL346 (in a ratio of 50:1) compared with the control group. Specifically, the level increased up to 29.8-fold, reaching 5.07 ng/mL, while in the control group, it was only 0.17 ng/mL. This increase was directly proportional to the amount of KBL346 used. Moreover, KBL346 caused a concentration-dependent increase in the expressions of iNOS and COX-2, as shown in Figure 2d.

### 2.3. KBL346 Increased the Production of Cytokines by Macrophages

As innate immune cells, macrophages have the ability to eliminate pathogens via phagocytosis. Additionally, they secrete several inflammatory mediators that activate and recruit other immune cells. Cytokines and chemokines are the most important of these mediators and they act as hormones to signal to other immune cells. Toll-like receptors (TLRs) are one type of pathogen recognition receptor (PRR) that activates signal transduction, leading to the secretion of inflammatory cytokines, such as TNF-α, IL-1β, and IL-6, as well as antiviral cytokines, such as type 1 interferon.

**Figure 3:**
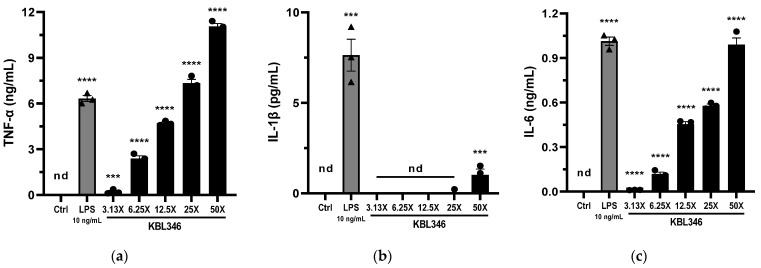
Effects of KBL346 on production of cytokines by macrophages. KBL346 and RAW264.7 cells were cocultured at ratios from 3.13:1 to 100:1. As a positive control group, treatment with 10 ng/mL of lipopolysaccharide (LPS) was used, and the control group (ctrl) was not treated with either LPS or bacteria. The level of immunomodulatory cytokines ((**a**) TNF-α; (**b**) IL-1β; (**c**) IL6) was measured in the culture supernatant using an ELISA kit. Data are expressed as the mean ± SD (*n* = 3). nd, not detected; * significant difference compared with control group (*** *p* < 0.001, **** *p* < 0.0001).

To determine the secretion of proinflammatory cytokines by RAW264.7 cells following exposure to KBL346, the levels of cytokines in the culture supernatant were measured via ELISAs. The results, shown in Figure 3, indicate that KBL346 increased the secretion of TNF-α and IL-6 in a concentration-dependent manner (Figure 3a,c). However, macrophages did not secrete a significant amount of IL-1β until exposed to 50 KBL346 bacteria per cell (Figure 3b).

### 2.4. KBL346 Activated Immune Signaling Pathways and Increased Expression of Their Target Genes in Macrophages

**Figure 4:**
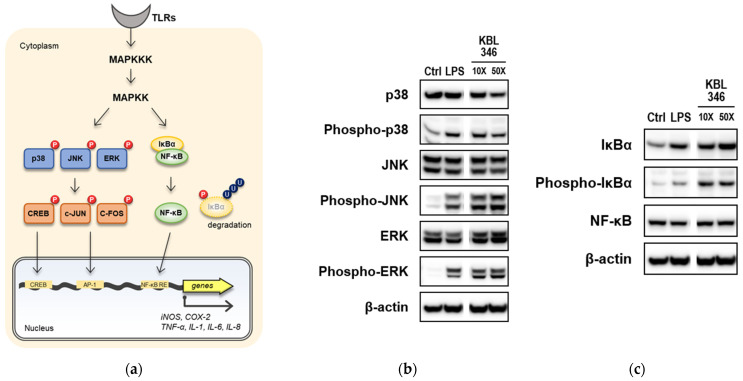
Effects of KBL346 on immune signaling pathways in macrophage. (**a**) Schematic representation of cellular signaling pathway of Toll-like receptors (TLRs) in macrophages. (**b**,**c**) After coculture of KBL346 and RAW264.7 cells at ratios of 10:1 and 100:1, phosphorylation and expression of mitogen-activated protein kinase (MAPK; blue in (**a**)) in MAPK signaling pathway or nuclear factor-κB (NF-κB; green in (**a**)) in the NF-κB signaling pathway were determined using immunochemical method. As a positive control group, treatment with 10 ng/mL concentration of LPS was used. β-actin was used as an internal loading control.

We conducted an analysis of the expression and phosphorylation of various kinases involved in different signaling pathways (Figure 4a). This was done to identify the mechanisms responsible for the immunostimulatory effects of KBL346. Macrophages, which have Toll-like receptors (TLRs) on their membranes and NOD-like receptors (NLRs) in their cytoplasm, express several pattern recognition receptors (PRRs). Specific receptors recognize different pathogen-associated molecular patterns (PAMPs). Among the PRRs expressed by macrophages, TLRs have been the focus of many studies on the intracellular response to their stimulation. The formation of ligand-induced TLR polymers and differences in adapter proteins according to the composition of these polymers have been studied. However, despite the different intracellular signaling processes, most studies suggest that TLRs activate either AP-1 family transcription factors (c-Jun, c-Fos, etc.) or the transcription factor NF-κB [19]. Therefore, we investigated the expression and phosphorylation of kinases involved in the signaling pathways that activate each family of transcription factors (Figure 4b,c).

When kinases within the MAPK signaling pathway (p38, JNK, and ERK) involved in the activation of AP-1 family transcription factors were examined, we found no significant differences in the expression of non-phosphorylated proteins compared with the control group. However, the level of phosphorylated protein (i.e., the activated form) increased significantly after treatment with KBL346 (Figure 4b). In the NF-kB signaling pathway, IkBα inhibits the activity of NF-kB by forming a complex with it. We found that IkBα was phosphorylated after KBL346 treatment, thereby allowing it to dissociate from NF-kB (Figure 4c). However, despite the increased amounts of phosphorylated IkBα, the level of NF-kB inside the cells did not increase significantly above the levels in the control group.

### 2.5. KBL346 Protected against Spleen and Body Weight Changes Decreased by Cyclophosphamide

**Figure 5:**
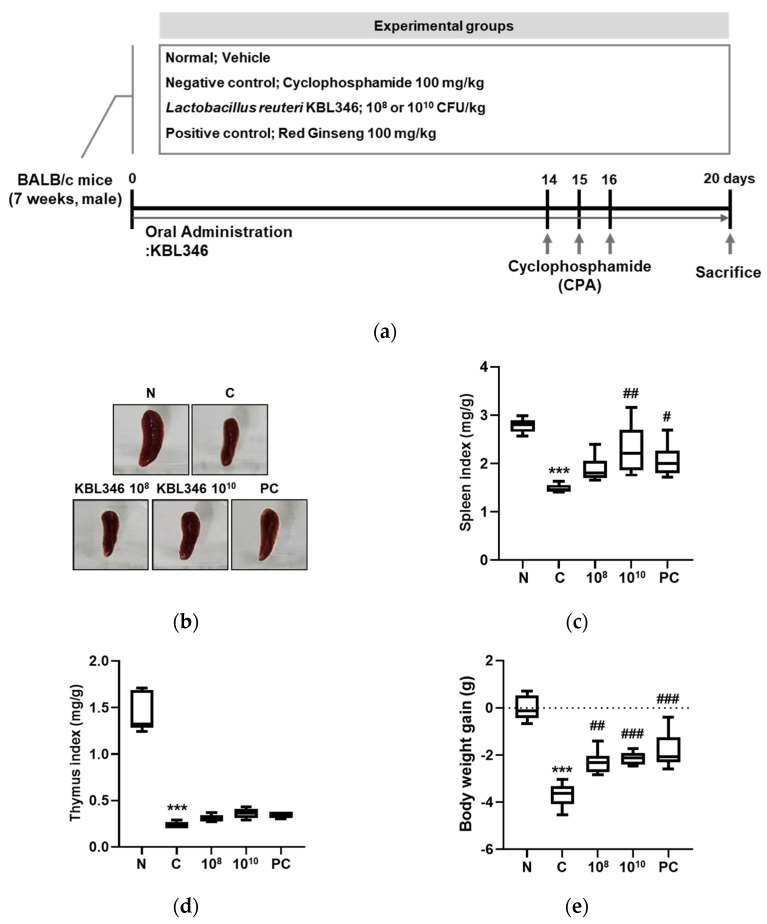
Effect of KBL346 on immune-enhancing activity in CPA-treated BALB/c mice. (**a**) Experimental design and timeline of in vivo study; (**b**,**c**) spleen size and spleen index; (**d**) thymus index; (**e**) body weight after 20 days of KBL346 administration. N, naïve mouse; C, CPA (100 mg/kg)-treated mouse; 10^8^, CPA-treated mouse administered with 10^8^ CFU/kg of KBL346; 10^10^, CPA-treated mouse administered with 10^10^ CFU/kg of KBL346; PC, positive control group administered with red ginseng (100 mg/kg). * Significant difference compared with the naïve group (*** *p* < 0.001). ^#^ Significant difference compared with the CPA-treated control group (^#^ *p* < 0.05, ^##^ *p* < 0.01, ^###^ *p* < 0.001).

To evaluate the immunostimulatory effect of KBL346 on physiological conditions, cyclophosphamide-induced immunosuppressed mice were used (Figure 5a). The effect of KBL346 was observed on the spleen size, body weight, thymus, and spleen index. The spleen size was significantly reduced by cyclophosphamide administration in the negative control groups in comparison with that of the normal group. However, KBL346 at 10^10^ CFU/kg dosage increased the spleen size, which was reduced using cyclophosphamide (Figure 5b). As shown in Figure 5c, KBL346 at 10^10^ CFU/kg significantly raised the spleen index by approximately 1.5-fold in comparison with that of the cyclophosphamide group, in which this improvement was superior to that of the red ginseng administration positive control group. However, the thymus index did not show significant changes in the KBL346 at both 10^8^ or 10^10^ CFU/kg dosage or red ginseng positive control group compared with the cyclophosphamide group (Figure 5d). The body weight was improved by KBL346 at both 10^8^ or 10^10^ CFU/kg similar to the red ginseng positive control group (Figure 5e). The results indicate that KBL346 had beneficial effects on immune organs and body weight change.

### 2.6. KBL346 Alleviated Spleen Tissue Damage Induced by Cyclophosphamide

**Figure 6:**
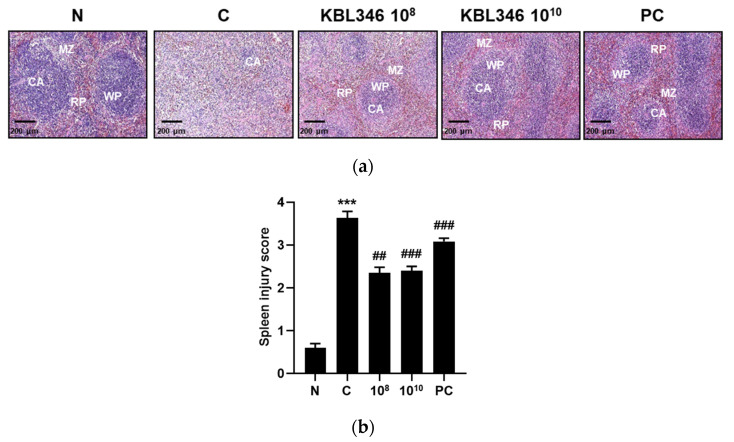
Effect of KBL346 on the restoration of structural disorganization (**a**) and injury score (**b**) of spleen in CPA-induced immunosuppressed mice. RP, red pulp; WP, white pulp; MZ, marginal zone; CA, central arteriole. N, naïve mouse; C, CPA (100 mg/kg)-treated mouse; 10^8^, CPA-treated mouse administered with 10^8^ CFU/kg of KBL346; 10^10^, CPA-treated mouse administered with 10^10^ CFU/kg of KBL346; PC, positive control group administered with red ginseng (100 mg/kg). * Significant difference compared with the naïve group (*** *p* < 0.001). ^#^ Significant difference compared with the CPA-treated control group (^##^ *p* < 0.001, ^###^ *p* < 0.001).

The spleen is an immunological organ and, therefore, structural damage may cause an increased risk of infection [20]. Cyclophosphamide-treated mice showed structural disorganization of the spleen compartments in comparison with that of the normal group. The blood-containing red pulp (RP), lymphocyte-containing white pulp (WP), and macrophage-containing marginal zone (MZ) were invisible. However, the spleen organization was restored by KBL346 at both 10^8^ or 10^10^ CFU/kg dosage, which was similar to that of the red ginseng administration positive control group (Figure 6a). In parallel with the spleen structural changes, spleen injury scores were significantly alleviated by KBL346 at both the 10^8^ or 10^10^ CFU/kg dosages by approximately 1.5-fold (Figure 6b). The results indicate that KBL345 potentially protected spleen function by restoring the spleen structural compartments.

### 2.7. KBL346 Enhanced Blood Cell Counts Decreased by Cyclophosphamide

**Figure 7:**
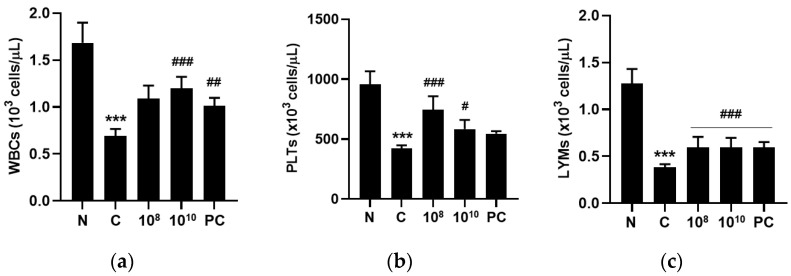

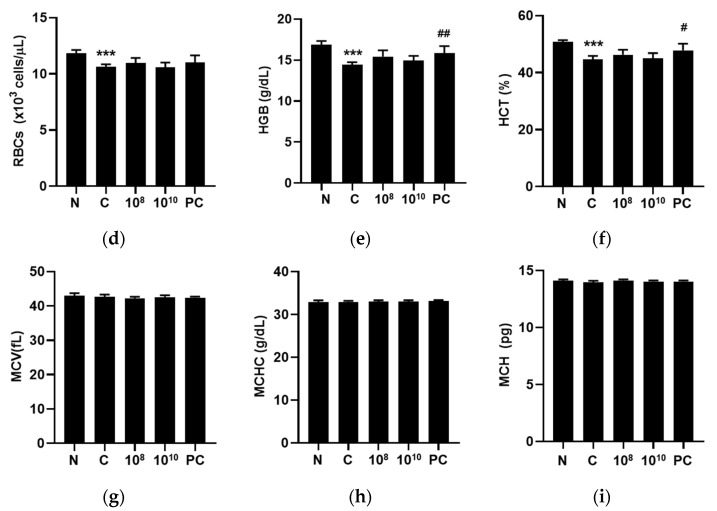
Changes in immunological blood cell profile by KBL346 in CPA-induced immunosuppressed mice. (**a**) WBCs, white blood cells; (**b**) PLTs, platelets; (**c**) LYMs, lymphocyte; (**d**) RBCs, red blood cell; (**e**) HGB, hemoglobin; (**f**) HCT, hematocrit; (**g**) MCV, mean corpuscle volume; (**h**) MCHC, mean corpuscle hemoglobin concentration; (**i**) MCH, mean corpuscle hemoglobin. N, naïve mouse; C, CPA (100 mg/kg)-treated mouse; 10^8^, CPA-treated mouse administered with 10^8^ CFU/kg of KBL346; 10^10^, CPA-treated mouse administered with 10^10^ CFU/kg of KBL346; PC, positive control group administered with red ginseng (100 mg/kg). * Significant difference compared with the naïve group (*** *p* < 0.001). ^#^ Significant difference compared with the CPA-treated control group (^#^ *p* < 0.05, ^##^ *p* < 0.01, ^###^ *p* < 0.001).

Cyclophosphamide administration induced a significant decrease in the immunological blood cell numbers. Decreased numbers of blood cells were found for white blood cells (WBCs), platelets (PLTs), lymphocytes (LYMs), red blood cells (RBCs), hemoglobins (HGBs), and hematocrits (HCTs). However, KBL346 at mostly the 10^10^ CFU/kg dosage elevated WBC, PLT, and LYM counts that were similar or superior to those of the red ginseng administration positive control group (Figure 7). The results show that KBL345 potentially had immunostimulatory effects by restoring immunological peripheral cells.

### 2.8. KBL346 Promoted Lymphocyte Proliferation and NK Cell Activity

Natural killer (NK) cells’ activity reflects the ability to produce immunological cytokines [21] and T and B lymphocytes are responsible for adaptive immune response. 

**Figure 8:**
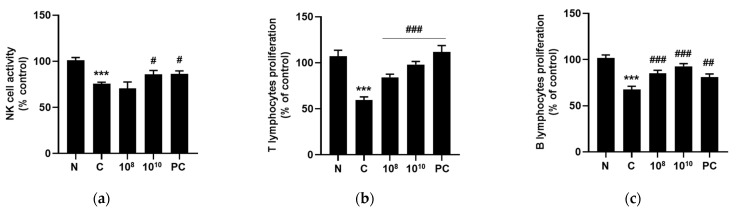
Effects of KBL346 on NK cell activity (**a**) and T cell (**b**) and B cell (**c**) proliferation in CPA-induced immunosuppressed mice. N, naïve mouse; C, CPA (100 mg/kg)-treated mouse; 10^8^, CPA-treated mouse administered with 10^8^ CFU/kg of KBL346; 10^10^, CPA-treated mouse administered with 10^10^ CFU/kg of KBL346; PC, positive control group administered with red ginseng (100 mg/kg). * Significant difference compared with the naïve group (*** *p* < 0.001). ^#^ Significant difference compared with the CPA-treated control group (^#^ *p* < 0.05, ^##^ *p* < 0.01, ^###^ *p* < 0.001).

Cyclophosphamide administration notably decreased the NK cell activity (1.5-fold) and T and B lymphocyte proliferation (2-fold and 1.5-fold, respectively). However, KBL346 administration at 10^10^ CFU/kg dosage effectively restored NK cell activity (*p* < 0.0001), as measured by LDH release levels in NK cells reacting with YAC-1 cells. The improved NK cell activity was similar to that of the red ginseng positive control group (Figure 8a). And KBL346 at both 10^8^ or 10^10^ CFU/kg dosage restored T lymphocyte (*p* < 0.001) and B lymphocyte (*p* < 0.0001) proliferation, as detected in splenocytes with concanavalin A (1 μg/mL) and LPS (1 μg/mL) for T cell and B cell proliferation, respectively (Figure 8b,c). This level of protection was similar to that of the red ginseng positive control group. The results indicated that KBL346 remarkably promoted immunity by inducing NK cell activity and T and B lymphocyte proliferation.

### 2.9. KBL346 Elevated CD4^+^ and CD8^+^ T Cell Numbers in Splenocytes Decreased by Cyclophosphamide

**Figure 9:**
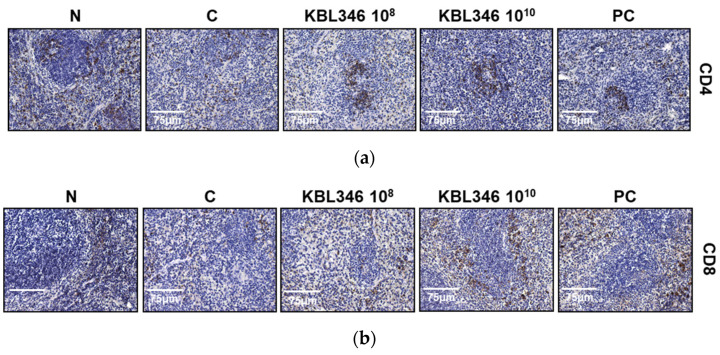

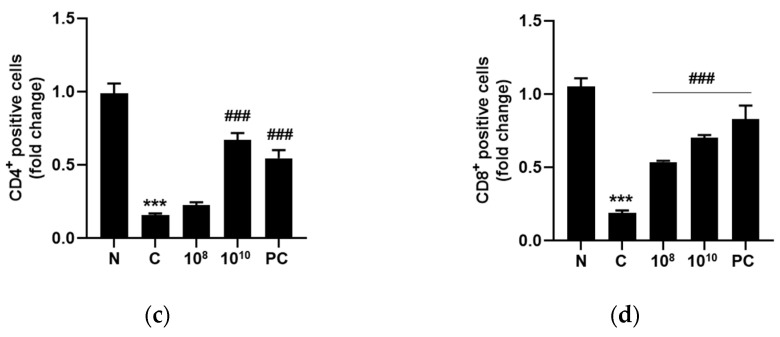
Restoration of CD4- and CD8-positive T lymphocytes in the spleen of CPA-induced immunosuppressed mice. (**a**,**b**) Representative immunohistochemical staining images and (**c**,**d**) fold changes of CD4^+^ and CD8^+^ cells in CPA-treated mouse spleen. Scale bar: 75 μm. N, naïve mouse; C, CPA (100 mg/kg)-treated mouse; 10^8^, CPA-treated mouse administered with 10^8^ CFU/kg of KBL346; 10^10^, CPA-treated mouse administered with 10^10^ CFU/kg of KBL346; PC, positive control group administered with red ginseng (100 mg/kg). * Significant difference compared with the naïve group (*** *p* < 0.001). ^#^ Significant difference compared with the CPA-treated control group (^###^ *p* < 0.001).

CD8^+^ T lymphocytes and CD8^+^ T lymphocyte-induced CD4^+^ cells play a major role in cellular-mediated immune responses to foreign antigen [22]. Therefore, changes in counts of CD4^+^- and CD8^+^-positive cells in the spleen were evaluated using immunohistochemistry. In splenocytes, cyclophosphamide significantly decreased the numbers of both CD4^+^- and CD8^+^-positive T cells. However, KBL346 at 10^8^ CFU/kg dosage remarkably enhanced cell counts of CD4^+^-positive T lymphocytes (*p* < 0.0001) superior to that of the red ginseng administration positive control group (Figure 9a,c). In addition, KBL346 at both the 10^8^ and 10^10^ CFU/kg dosages significantly restored the cell counts of CD8^+^-positive T lymphocytes (Figure 9b,d). The results show that KBL346 promoted cell counts of CD4^+^ and CD8^+^ T lymphocytes that could increase immunity.

### 2.10. KBL346 Stimulated Secretion of Immune-Associated Cytokines

**Figure 10:**
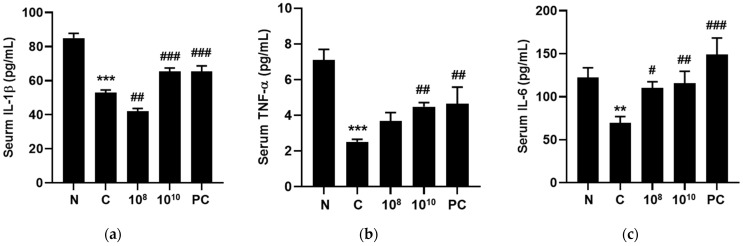

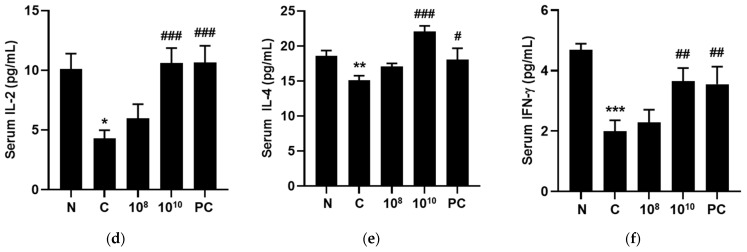
Effect of KBL346 on serum cytokine levels in CPA-induced immunosuppressed mice. (**a**) IL-1β, (**b**) TNF-α, (**c**) IL-6, (**d**) IL-2, (**e**) IL-4, and (**f**) IFN-γ levels in serum. N, naïve mouse; C, CPA (100 mg/kg)-treated mouse; 10^8^, CPA-treated mouse administered with 10^8^ CFU/kg of KBL346; 10^10^, CPA-treated mouse administered with 10^10^ CFU/kg of KBL346; PC, positive control group administered with red ginseng (100 mg/kg). * Significant difference compared with the naïve group (* *p* < 0.05, ** *p* < 0.01, *** *p* < 0.001). ^#^ Significant difference compared with the CPA-treated control group (^#^ *p* < 0.05, ^##^ *p* < 0.01, ^###^ *p* < 0.001).

Cytokines are essential proteins involved in generating and regulating immune cells. In an attempt to study the effect of KBL346 on immune-associated cytokines, the levels of Interleukin (IL)-1β, Tumor Necrosis Factor (TNF)-α, IL-6, IL-2, IL-4, and Interferon (IFN)-γ were measured in mouse blood serum using ELISA. As depicted in Figure 10, the administration of cyclophosphamide significantly reduced these immune-related cytokine levels. However, KBL346 at both dosages of 10^8^ and 10^10^ CFU/kg significantly restored the serum levels of these cytokines, specifically IL-1β and IL-6. Moreover, at a dosage of 10^10^ CFU/kg, KBL346 significantly increased the production of TNF-α, IL-2, IL-4, and IFN-γ. The increase in cytokine by KBL346 was comparable with that of the red ginseng administration positive control group. These results indicate that KBL346 has the potential to stimulate immunity by increasing immune-related cytokines.

### 2.11. KBL346 Stimulated Secretion of Immune-Related Immunoglobulins

**Figure 11:**
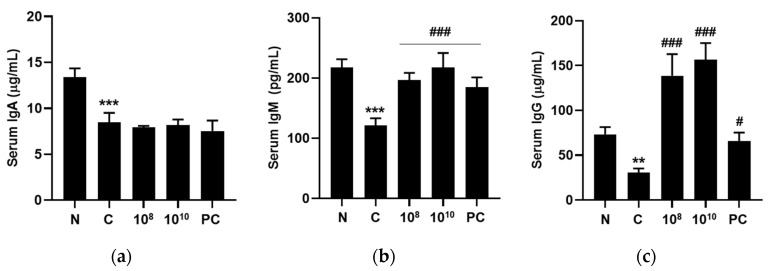
Effect of KBL346 on serum immunoglobulin levels in CPA-induced immunosuppressed mice. (**a**) IgA, (**b**) IgM, and (**c**) IgG levels in serum. N, naïve mouse; C, CPA (100 mg/kg)-treated mouse; 10^8^, CPA-treated mouse administered with 10^8^ CFU/kg of KBL346; 10^10^, CPA-treated mouse administered with 10^10^ CFU/kg of KBL346; PC, positive control group administered with red ginseng (100 mg/kg). * Significant difference compared with the naïve group (** *p* < 0.01, *** *p* < 0.001). ^#^ Significant difference compared with the CPA-treated control group (^#^ *p* < 0.05, ^###^ *p* < 0.001).

Immunoglobulins (Igs) are a type of glycoprotein that plays a crucial role in maintaining the immune system. In order to examine immunoglobulins, blood serum levels of IgA, IgM, and IgG were measured using ELISA (Figure 11). Cyclophosphamide significantly decreased serum levels of IgA, IgM, and IgG. However, the administration of KBL346 at both 10^8^ and 10^10^ CFU/kg dosages restored the levels of IgM (*p* < 0.0001) and IgG (*p* < 0.0001) to levels superior to those of the red ginseng administration positive control group. The level of IgA was not significantly changed by either KBL346 at 10^8^ or 10^10^ CFU/kg dosage or the red ginseng administration positive control group. These results show that KBL346 improved immunity by increasing the levels of immunological Ig.

### 2.12. Safety Profile of KBL346

**Figure 12:**
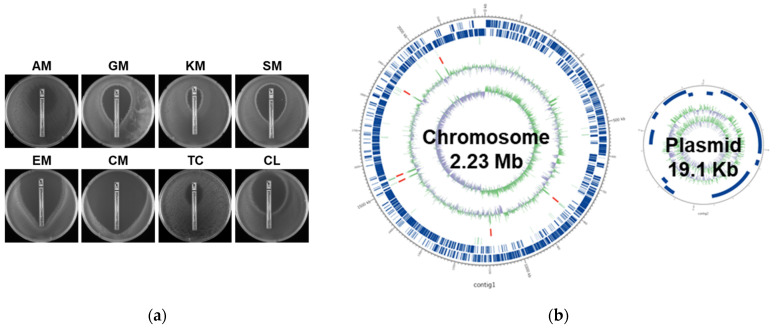

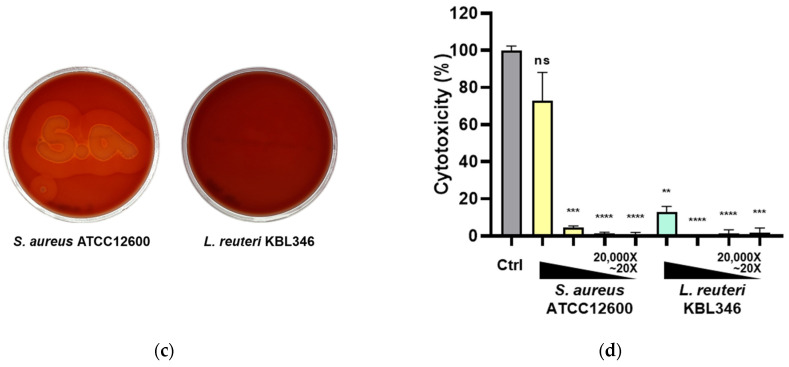
Safety assessment of KBL346 as a probiotic strain. (**a**) Images from antibiotic resistance test using E-strip. AM, ampicillin; GM, gentamicin; KM, kanamycin; SM, streptomycin; EM, erythromycin; CM, clindamycin; TC, tetracyclin; CL, chloramphenicol. (**b**) Genome Atlas view of the chromosome and plasmid in *L. reuteri* KBL346. (**c**) Hemolytic activity analysis of *S. aureus* ATCC12600 (**left**) and KBL346 (**right**) measured in BHI broth containing blood. (**d**) Cytotoxicity of Caco-2 cells measured using LDH activity assay after the treatment of ATCC12600 and KBL346 (Caco-2 cells:bacteria = 20,000:1). ns, not significant compared with the control group (*p* > 0.05); * significant difference compared with the control group (** *p* < 0.01, *** *p* < 0.001, **** *p* < 0.0001).

We conducted tests to determine the safety of using KBL346 as a probiotic. We examined its antibiotic resistance, hemolytic activity, cytotoxicity, and metabolic characteristics. The E-strip test showed that KBL346 is susceptible to eight antibiotics, which is within the EFSA breakpoint (as shown in Table 1 and Figure 12a). Additionally, no antibiotic resistance genes were found in either the 2.23 Mb chromosome or the 19.1 kb plasmid (Table 2 and Figure 12b). Unlike *Staphylococcus aureus* ATCC12600, KBL346 is identified as a γ-hemolysis bacterium that does not show hemolytic activity (as shown in Figure 12c). To confirm cytotoxicity against Caco-2 cells, which is a human intestinal epithelial cell line, we tested LDH activity, and the results showed that KBL346 caused no significant cytotoxicity to any of the treated groups (as shown in Figure 12d). When *S. aureus* ATCC12600 was treated with 20,000 times the number of cells, the cytotoxicity was similar to that of the positive control. However, KBL346 caused no significant cytotoxicity even when treated with a high number of cells.

Finally, we examined metabolic characteristics, BSH activity, and D-lactate production. BSH activity was confirmed by streaking the KBL346 strain on MRS agar medium containing taurodeoxycholic acid (TDCA), which is a bile acid. If the strain has BSH activity, the colonies appear as opaque white circles; however, in the case of KBL346, no opaque white colonies were formed, indicating that the strain was negative for BSH activity. To confirm whether KBL346 produces D-lactate, D-lactate concentrations in the culture supernatant comprising two media (BHI and MRS) were measured in an ELISA. Although there was a difference in the amount produced, D-lactate was detected in both media (0.16 mM in BHI medium; 7.56 ± 0.17 mM in MRS medium). Humans cannot metabolize D-lactate; therefore, if orally ingested probiotics contain DL-lactate racemase, which converts L-lactate to D-lactate, D-lactate accumulates and causes acidosis. Since the accumulation of D-lactate is a problem for newborns, children, and patients with congenital short bowel syndrome, it can only be used in conjunction with a warning statement according to the WHO/FAO guidelines.

## 3. Discussion and Conclusions

In this study, we investigated the immunostimulatory properties of *L. reuteri* KBL346 in both in vitro macrophage cells and in vivo CPA-induced immune-suppressed BALB/c mice. Additionally, we evaluated the safety profile of KBL346 using a variety of tests based on the EFSA guidelines. 

*Lactobacillus* spp. have indeed gained significant attention in clinical and medical settings due to their safety, reliability, and promising performance in a range of health conditions [23]. However, it is important to note that the effects of *Lactobacillus* on the immune system can be strain specific, and individual responses may vary. For over 30 years, *L. rhamnosus* GG (LGG) has been reported to be effective in treating various diseases, including gastrointestinal, respiratory, cardiovascular, metabolic, and autoimmune diseases [24]. LGG has been available commercially for a long time and is considered a reliable strain due to its functionality. However, it may not work effectively for everyone, or it may not address specific health concerns, such as its immunostimulatory efficacy in a cyclophosphamide-induced condition. The effectiveness of probiotics can be influenced by various factors, including their metabolic capacity, the composition of bioactive molecules they produce, and the individual differences in the gut microbiome of the host. Therefore, it is still important and necessary to discover more effective and specific probiotic strains that cater to each individual’s health goals and status. This will help to take a more personalized and informed approach to probiotic use.

Immune suppression, whether due to medical conditions, medications, or other factors, can significantly increase the risk of developing infections, reactivating latent infections, and experiencing prolonged illness. It can also increase the risk of developing cancer and reduce the effectiveness of vaccines [25,26]. Cyclophosphamide (CPA) has been a widely used alkylating agent for more than 40 years for the treatment of cancer and as an immunosuppressive agent for the treatment of autoimmune and immune-mediated diseases [27]. One of the main side effects of CPA is the development of myelosuppression, leading to increased susceptibility to infections due to the reduction in immune cells [28]. Our research showed that when we gave KBL346 to mice that were immunosuppressed and treated with CPA orally, it significantly improved the condition of their spleen tissue damage, blood cell counts, immune-associated cytokine and immunoglobulin secretion, and T and B lymphocyte proliferation and activities, as well as NK cell activity. Treatment with KBL346 also increased the phagocytic activity of macrophages and stimulated the secretion of TNF-α, IL-1β, IL-6, NO, and PGE_2_ via the TLR-MAPK signaling pathway. In particular, NO can act as an important intracellular signal; however, it also can cause NO-mediated cytotoxicity, which is related to the inflammatory function of M1-type macrophages and is suppressed via intracellular factors, such as MRP1 and GSTP1 [29,30]. The treatment using KBL346 controlled the excessive production of NO in comparison to PGE_2_ and showed no cytotoxic effect on cells. 

Several strains of *Lactobacillus* species, including *L. rhamnosus* NCU011054 [9], *L. rhamnosus* CRL1506 [31], *L. reuteri* PSC102 [32], *L. plantarum* Lp2v [10], *L. plantarum* 20655 [11], *L. plantarum* NCU116 [12], and *L. acidophilus* LA85 [13], showed immune-boosting effects in a CPA-induced immunosuppressed model. *L. casei* CRL431 and *L. paracasei* CNCM I-1518 increase immune-related cytokines, such as IL-6 and macrophage chemoattractant protein 1, and mediate immune stimulation through Toll-like receptors [33,34]. *L. acidophilus* LA85 ameliorates immunosuppression by modulating Notch and TLR4/NF-κB signal pathways [13].

*L. reuteri* is a probiotic species widely used as a dietary supplement. It was reported to modulate insulin sensitivity, glucose homeostasis, and systemic inflammation, and alleviate the symptoms of various diseases, such as obesity, hepatic disorders, brain disorders, infant diarrhea, inflammatory bowel disease, cystic fibrosis, and autoimmune diseases (reviewed in [15]). However, only a heat-killed *L. reuteri* PSC102 strain has been reported to have immunostimulatory efficacy among the diverse *L. reuteri* strains. Although the anti-inflammatory and regulatory mechanisms of *L. reuteri* and other lactobacilli are relatively well-specified, the molecular mechanisms of immune-boosting by the same species need further study. 

In conclusion, *L. reuteri* KBL346 can effectively stimulate the innate and adaptive immune systems in both in vitro and in vivo systems without any safety concerns. As a result, it could be used as a potent immunostimulatory probiotic strain for enhancing immunity. However, the molecular mechanism of how *L. reuteri* KBL346 differs from other strains in modulating the immune function of both gene and protein expression levels requires further investigation. A longitudinal human study with the tracking of the absorption–distribution–metabolism–excretion properties of KBL346 can also provide valuable data for KBL346 as a useful probiotic strain.

## 4. Materials and Methods

### 4.1. Isolation and Identification of the L. reuteri KBL346

*L. reuteri* KBL346 (hereafter referred to as KBL346) was isolated from a fecal sample from a 3-month-old infant (provided by SAMSUNG MEDICAL CENTER (SMC); Seoul, Republic of Korea). After culturing the single colony, the genomic DNA was extracted and analyzed. 16S ribosomal RNA (rRNA) gene sequencing and whole-genome sequencing (both performed by Macrogen Inc., Seoul, Republic of Korea) revealed that the isolated strain KBL346 showed the highest homology with *L. reuteri*. This strain was deposited in the Korean Collection for Type Culture (KCTC) under registration number KCTC 15268BP.

### 4.2. Preparation of Bacterial Samples

Lyophilized bacteria were used for the efficacy test (provided by KoBioLabs, Seoul, Republic of Korea). Briefly, 1 g of lyophilized KBL346 powder was reconstituted in 9 mL of sterile phosphate-buffered saline (PBS) buffer (Gibco, Grand Island, NY, USA, 10010-023), and the bacteria were recovered by centrifugation at 12,000× *g* for 5 min at 4 °C. The bacterial cell pellet was washed twice with PBS buffer to remove additives, such as cryoprotectants, and adjusted to the appropriate density in Dulbecco’s modified Eagle medium (DMEM, Gibco, 10313-021) or Roswell Park Memorial Institute 1640 (RPMI 1640, Gibco, 22400-089) medium. Otherwise, live bacteria were used for the safety test. KBL346 was inoculated onto a de Man, Rogosa, and Sharpe (MRS, BD Difco, Franklin Lakes, NJ, USA, 288130) medium or Brain Heart Infusion (BHI, BD Difco, 237500) medium, and cultured for 16–24 h at 37 °C under anaerobic conditions. As a positive control, *Staphylococcus aureus* ATCC12600 was inoculated into a Tryptic soy broth (TSB, BD Difco, 211825) medium and cultured for 16–24 h at 37 °C under aerobic conditions. The CFU for each bacterial sample was quantified using a 10-fold serial dilution and the plate-count method of MFDS.

### 4.3. Cell Culture

The murine macrophage cell line, RAW264.7 was cultured at 37 °C/5% CO_2_ in DMEM containing 10% inactivated fetal bovine serum (FBS, WELGENE, Gyeongsan, Republic of Korea, S 001-01), 100 U/mL penicillin, and 100 mg/mL streptomycin (Gibco, 15140-122). The human colon epithelial cell line, Caco-2 was cultured at 37 °C/5% CO_2_ in DMEM/F-12 (Gibco, 11320033) containing 20% inactivated FBS, 1% MEM nonessential amino acid solution (Gibco, 11140-050), 10 mM HEPES (Gibco, 15630-080), 0.1% sodium bicarbonate solution (Gibco, 25080-094), 10 μg/mL of gentamicin, 100 U/mL penicillin, and 100 mg/mL streptomycin. When the cells were about 80% confluent, they were detached from the culture dish and used for experiments.

### 4.4. Coculture Conditions

RAW264.7 or Caco-2 cells were seeded for each experiment: 5 × 10^4^ cells/well (200 μL, 96-well plates; cell viability, nitric oxide level, cytokine level, PGE_2_ level, LDH assay), 8 × 10^5^ cells/well (1 mL, 6-well plates; phagocytosis assay), 2 × 10^6^ cells/dish (10 mL, 100 mm cell culture dish; protein expression). All plates were incubated for 24 h at 37 °C/5% CO_2_. Next, the KBL346 strain was added to the cultures and incubated at 37 °C for 24 h. After coculture, the culture supernatant or cell pellet was analyzed as required.

### 4.5. Evaluation of Macrophage Phagocytic Activity

RAW264.7 cells were cocultured with KBL346 at a ratio of 1:5. Next, RAW264.7 cells were washed twice with PBS buffer and then treated with the yeast *Candida albicans* MYA-4788 at an MOI (multiplicity of infection) of 10 at 37 °C for 1 h. After the *C. albicans* treatment, RAW264.7 cells were washed twice with PBS, fixed for 1 h with 2.5% glutaraldehyde, and stained for 20 min with 0.5% methylene blue. All cells were detached from the culture plate using a cell scraper and observed under an optical microscope. If more than one *C. albicans* was engulfed in a macrophage, it was considered to have triggered phagocytosis. Phagocytic activity was calculated as follows: Phagocytic activity (%) = (Number of phagocytosis-triggering cells/Total number of observed macrophage cells) × 100.

### 4.6. Measurement of Cell Viability

The viability of RAW264.7 cells was assessed using an MTT (3-(4,5-dimethylthiazol-2-yl)-2,5-diphenyltetrazolium bromide) assay kit (Promega, Madison, WI, USA, G4000). After being cocultured with KBL346, the RAW264.7 cells were washed twice with PBS, and the cell viability was assessed using cell pellets, as described in the manufacturer’s protocol. A sample treated with neither bacteria nor LPS was used as a negative control. In the case of the LPS- or KBL346-treated groups, viability was compared with that of the negative control.

### 4.7. Measurement of NO, PGE_2_, and Cytokine Levels

The levels of NO, PGE_2_, and cytokines (TNF-α, IL-1β, IL-6) in the culture supernatant of RAW264.7 cells cocultured with KBL346 were measured. The amount of NO produced by macrophages was analyzed using the Griess reaction. The Griess reagent was prepared by mixing Griess reagent A (1% sulfanilamide in 5% phosphoric acid) (sulfanilamide, Sigma-Aldrich Co., St. Louis, MO, USA, 33626-100G) (phosphoric acid, Sigma-Aldrich Co., 49685-100ML) and Griess reagent B (0.1% N-(1-naphthyl) ethylenediamine dihydrochloride in DW) (Sigma-Aldrich Co., 33461-25G) at a 1:1 ratio. Absorbance was measured at 540 nm after mixing equal amounts of the sample and Griess reagent and incubating in the dark for 20 min. The concentration of NO was calculated from a standard calibration curve obtained using sodium nitrite (NaNO_2_) (Sigma-Aldrich Co., 7632-00-0). The levels of PGE_2_ and cytokines in the cocultured cell supernatant were measured using ELISA kits: PGE_2_ (Cayman CHEMICAL, Ann Arbor, Michigan, USA, 514010), TNF-α (558534), IL-6 (555240) (BD Biosciences, San Jose, CA, USA), and IL-1β/IL-1F2 (R&D SYSTEMS, Minneapolis, MN, USA, DY401).

### 4.8. Western Blot

Changes in expression and phosphorylation levels of immune-related proteins in macrophages were examined immunochemically using specific antibodies. After coculture with KBL346, RAW264.7 cells were lysed in radioimmunoprecipitation assay (RIPA) buffer (Thermo Fisher Scientific, Waltham, MA, USA, 89900), and the supernatant was separated by centrifugation. Total cellular proteins in the supernatant were quantified in a bicinchoninic acid (BCA) assay (Thermo Fisher Scientific, 23227). Proteins were separated on sodium dodecyl sulfate-polyacrylamide gels (SDS-PAGE). After the transfer of the proteins to a polyvinylidene difluoride (PVDF) membrane, the membrane was blocked for 1 h in a TBS-T solution containing 5% bovine serum albumin (BSA, Sigma-Aldrich Co., A2153). After blocking, the membranes were incubated with the target primary antibody (4 °C overnight) at the dilution recommended by each manufacturer. The following were the specific antibodies used for each biomarker: iNOS (Cayman, 160862), COX-2 (Cayman, 160106), Phospho-IκBα (2859S), IκBα (4812S), NF-κB p65 (8242S), Phospho-p38 MAPK (4511S), p38 MAPK (9212S), Phospho-ERK1/2 (4370S), ERK1/2 (4695S), Phospho-JNK (9251S), SAPK/JNK (9252S) (Cell Signaling Technology, Danvers, MA, USA), and anti-β-Actin (N-term) (Abfrontier, Seoul, Republic of Korea, LF-PA0207A). After washing three times with TBS-T, a horse radish peroxidase-conjugated secondary antibody was added at a dilution of 1:5000 and incubated for 1 h at room temperature. The blots were probed using an ECL solution and imaged by a ChemiDoc analyzer.

### 4.9. Animals

The effect of KBL346 on immune and hematopoietic function was evaluated in 7-week-old, male BALB/c mice (Samtako Bio Korea, Osan, Republic of Korea). The mice were maintained in standard laboratory conditions (temperature 20–25 °C, humidity 50–60%, and 12 h light–dark cycle). Mice were randomly allocated to five groups: normal (N), cyclophosphamide (CPA) (CPA, Sigma-Aldrich Co.), red ginseng positive control (PC), and KBL346 at low (10^8^ CFU/kg) and high (10^10^ CFU/kg) dosages, as described previously with some modification [35,36,37,38]. The total duration of the animal experiment was 20 days. Intraperitoneal administration of cyclophosphamide (100 mg/kg) was performed on the 14th, 15th, and 16th days. This study was carefully reviewed and approved by the Institutional Animal Care and Use Committee of Dong-eui University (approval no. A2022-018).

### 4.10. Collection of Blood Samples

At the end of the experiment, mice were sacrificed via carbon dioxide (CO_2_) inhalation and blood was collected via cardiac puncture. Collected blood was centrifuged at 2000 rpm and 4 °C to obtain blood serum for immune-associated biomarker analysis.

### 4.11. Hematological Analysis

Hematological parameters include white blood cell, platelet, lymphocyte, red blood cell, hematocrit, hemoglobin, mean corpuscular volume, mean corpuscular hemoglobin, and mean corpuscular hemoglobin concentration. These parameters were evaluated using a blood cell analyzer (Sysmex Corporation, Kobe City, Japan), as previously described [39].

### 4.12. Immune-Associated Marker Analysis

Serum contents of immunoglobulins and cytokines were measured using an enzyme-linked immunosorbent assay kit. The following were evaluated: Mouse/Rat-immunoglobulin immunoglobulin (Ig) M (IgM, ab215085), IgA (ab157717), IgG (ab151276) (Abcam Inc., Cambridge, UK), Mouse tumor necrosis factor-α (SMTA00B), interleukin (IL)-6 (M600B), and IL-1β (MLB00C) (R&D Systems, Minneapolis, MN, USA) conjugated antibodies, as described in the manufacturer’s protocol.

### 4.13. Splenocytes Isolation

The mice spleens were incubated in RPMI1640 medium (WelGENE) and homogenized using a 40 μm nylon cell strainer (BD Bioscience). These homogenates were centrifuged at 1000 rpm and 4 °C and red blood cells were removed using a red blood cell lysis buffer (Sigma-Aldrich Co., St. Louis, MO, USA) and added to RPMI 1640 medium for cell growth.

### 4.14. T and B Lymphocytes Proliferation Analysis

The mice splenocytes were obtained and seeded into a 96-well plate and lipopolysaccharides (1 μg/mL) (LPS, Sigma-Aldrich Co.) and concanavalin A (1 μg/mL) (Con A, Sigma-Aldrich Co.) were treated for B lymphocyte and T lymphocyte proliferation. The proliferation was evaluated using a CCK-8 assay (Abcam Inc., Cambridge, UK), as described in the manufacturer’s protocol.

### 4.15. Natural Killer Cell Activity Measurement

The natural killer (NK) cells were obtained from splenocytes and were reacted with YAC-1 cells at 37 °C and 5% CO_2_ for 4 h. Then, the NK cell activity was measured using the lactate dehydrogenase (LDH) release level with a colorimetric L-LDH assay kit (Thermo Fisher Scientific), as described in the manufacturer’s protocol.

### 4.16. Lymphocyte Subpopulation Analysis

Numbers of CD4^+^ and CD8^+^ T cells were analyzed by staining splenocytes by FITC Rat Anti-Mouse CD8a and PE Rat Anti-Mouse CD4 for CD8 and CD4, respectively. After washing cells with PBS, counts of positive cells were analyzed using a flow cytometry analyzer (Accuri C6, BD Bioscience, Ann Arbor, MI, USA) at the Core-Facility Center for Tissue Regeneration, Dong-eui University.

### 4.17. Hematoxylin and Eosin (H&E) Staining

Histological detection of spleens was detected using H&E staining. The paraffin-embedded tissues were cut with a thickness of 4 μm into a slide specimen. The prepared slides were stained with H&E (Sigma-Aldrich Co.), as previously described [40].

### 4.18. Safety Profile of KBL346

The safety profile of KBL346 was analyzed in accordance with the “guidelines for safety assessment of probiotics” issued by the MFDS. The safety profile includes antibiotic resistance (minimum inhibitory concentration (MIC)), hemolytic activity, cytotoxicity (LDH activity test), and the metabolism of bile acids (bile salt hydrolase (BSH) activity) and D-lactate. Susceptibility to eight antibiotics (ampicillin, gentamicin, kanamycin, streptomycin, erythromycin, clindamycin, tetracycline, and chloramphenicol) was determined using a commercial E-test strip. Suspensions of bacterial culture with turbidity equivalent to McFarland standard 1 were swabbed evenly onto an LSM (LAB susceptibility test medium; 90% IST broth, 10% MRS broth, 1.8% agar) plate using a sterile cotton swab, and E-test strips were placed on the surface of the plates. After incubation at 37 °C for 24 h, antibiotic resistance was evaluated in accordance with the “guidelines for antibiotic resistance of the European Food Safety Authority (EFSA)”. The bacteria were streaked onto blood agar plates for the hemolytic activity test. Hemolytic activity was classified according to the pattern of the zones formed around the colony (clear zones: β-hemolysis, green-hued zones: α-hemolysis, no zones: γ-hemolysis). The cytotoxicity of KBL346 was evaluated in an LDH activity test using a Lactate Dehydrogenase Assay Kit. The cytotoxicity was calculated as follows: Cytotoxicity (%) = {(Test sample−Negative control)/(Positive contro−Negative control)} × 100. A positive control lysate was obtained by dissolving Caco-2 cells in a lysis buffer containing a surfactant. A sample treated with neither bacteria nor lysis buffer was used as a negative control. Bile salt metabolism was confirmed by streaking bacteria onto an MRS plate supplemented with 0.5% TDCA, and the activity of BSH was tested. If no opaque white colonies were formed around a single colony, it was regarded as having no BSH activity. D-lactate metabolism was confirmed by measuring the D-lactate levels in culture supernatant from bacteria using a D-lactate assay kit. *Staphylococcus aureus* ATCC12600 was used as a control strain for the hemolysis and cytotoxicity tests.

### 4.19. Statistical Analysis

All data are expressed as the mean ± standard deviation. Statistical analysis of the in vitro data was performed using an independent *t*-test (*p* < 0.05, *p* < 0.01, *p* < 0.001, or *p* < 0.0001) using GraphPad PRISM version 9 (GraphPad Software, San Diego, CA, USA). Statistical analysis of in vivo data was performed using one-way ANOVA of GraphPad prism^®^ Ver 5.0 (GraphPad Inc.). The *p* < 0.5 was significant via post hoc testing with Tukey’s test.

## Figures and Tables

**Table 1:** Minimum inhibitory concentration (MIC) of antibiotics and qualitative analysis of antibiotic resistance genes.

Antibiotic (mg/L)		AM	GM	KM	SM	EM	CM	TC	CL
Susceptibility	EFSA BP	2	8	64	64	1	1	16	4
	KBL346	2	0.38	12	4	0.023	<0.016	12	0.75
Resistance gene		n.d.	n.d.	n.d.	n.d.	n.d.	n.d.	n.d.	n.d.

Abbreviations: EFSA BP, European Food Safety Authority breakpoint; AM, ampicillin; GM, gentamicin; KM, kanamycin; SM, streptomycin; EM, erythromycin; CM, clindamycin; TC, tetracyclin; CL, chloramphenicol; n.d., not detected.

**Table 2:** Characteristics of the *L. reuteri* KBL346 genome.

Contig Name	Size (bp)	GC (%)	CDS	tRNA	rRNA
Contig 1	2,227,886	39.1	2151	69	18
Contig 2	19,058	36.9	17	0	0
Total	2,246,944	39.0	2168	69	18

Abbreviations: bp, base pairs; GC, guanine–cytosine; rRNA, ribosomal ribonucleic acid; tRNA, transfer ribonucleic acid; CDS, coding sequence.

## Data Availability

The data presented in this study are available on request from the corresponding author.
